# Analyzing Menton Deviation in Posteroanterior Cephalogram in Early Detection of Temporomandibular Disorder

**DOI:** 10.1155/2017/5604068

**Published:** 2017-08-06

**Authors:** Trelia Boel, Ervina Sofyanti, Erliera Sufarnap

**Affiliations:** ^1^Department of Dental Radiology, Faculty of Dentistry, University of Sumatera Utara, Medan, Indonesia; ^2^Department of Orthodontics, Faculty of Dentistry, University of Sumatera Utara, Medan, Indonesia

## Abstract

**Introduction:**

Some clinicians believed that mandibular deviation leads to facial asymmetry and it also had a correlation with temporomandibular disorders (TMDs). Posteroanterior (PA) cephalogram was widely reported as a regular record in treating facial asymmetry and craniofacial anomalies. The objective of this study was to analyze the relationship of menton deviation in PA cephalogram with temporomandibular disorders (TMDs) symptoms.

**Materials and Methods:**

TMJ function was initially screened based on TMD-DI questionnaire. PA cephalogram of volunteer subjects with TMDs (*n* = 37) and without TMDs (*n* = 33) with mean age of 21.61 ± 2.08 years was taken. The menton deviation was measured by the distance (mm) from menton point to midsagittal reference (MSR) horizontally, using software digitized measurement, and categorized as asymmetric if the value is greater than 3 mm. The prevalence and difference of menton deviation in both groups were evaluated by unpaired *t*-test.

**Result:**

The prevalence of symmetry group showed that 65.9% had no TMDs with mean of 1,815 ± 0,71 mm; in contrast, the prevalence of asymmetry group showed that 95.5% reported TMDs with mean of 3,159 ± 1,053 mm. There was a significant difference of menton deviation to TMDs (*p* = 0.000) in subjects with and without TMDs.

**Conclusion:**

There was a significant relationship of menton deviation in PA cephalogram with TMDs based on TMD-DI index.

## 1. Introduction

The relevance of temporomandibular disorders (TMDs) to malocclusion became a hot issue in recent years. TMDs are a collective complex term for a group of musculoskeletal and neuromuscular conditions which includes several clinical signs and symptoms involving the muscles of mastication, temporomandibular joint, and associated structures [1, 2]. The displacement of mandible can influence the modeling process of the TMJ, leading to asymmetry. Even though a small amount of asymmetry in the maxillofacial region is common, there is a critical threshold distance that is considered as asymmetric [3–6]. At the same time many authors have shown no or weak connection between orthodontics treatment and TMDs.

Malocclusion itself is a product of multiple factors that yields a significant influence on the patient's quality of life during craniomandibular growth and development even though, until aging, it can be treated by orthodontics or orthognathic surgery. The mandibular asymmetry is a major problem due to its effect on facial appearance directly, especially the chin area that represents the third lower facial part. There is an impact on the quality of life because functional problems that related to the role of temporomandibular joint are in the stomatognathic system and also affect the facial appearance, such as facial asymmetry [7, 8]. In daily practice, posteroanterior cephalogram is used widely in detection of asymmetry mandible that involved skeletal and dentoalveolar component [9]. Investigating the connection between morphologically anatomy landmarks with sign and symptom of TMDs has been a more interesting question than debating about malocclusion causes of TMDs or vice versa. Recent study reported sign and symptom of TMD as main risk factor in the occurrence of mandibulofacial asymmetry [10]. Asymmetry in mandibular usually results in a shift of the chin and 70% of patients with facial asymmetry and chin deviation presented structural and displacement asymmetry, while only 10% showed pure displacement asymmetry and facial askeletal asymmetry was reported to exist in patients with chin deviation [11, 12].

The aim of this study is to investigate the menton deviation that is presented at the chin position in a PA cephalogram. Early detection of TMDs symptom is performed by questionnaire, neglecting TMDs sign. Clarifying the relationship between menton deviation and TMDs symptoms is required to develop diagnosing and planning treatment of mandibular asymmetry.

## 2. Materials and Methods

All study volunteers who were female students of Dental Faculty of University of Sumatera Utara signed an informed consent form to participate in this study. This was a cross-sectional case-control study from March 2016 until August 2016 with 37 subjects with TMDs and 33 subjects without TMDs. An index as was showed in Table 1 was developed in Indonesia, called TMD diagnostic index (TMD-DI) as early detection of symptom was applied by the examiners in screening protocol of those volunteers [13].

The following inclusion criteria were used for volunteers participation in the study: (1) still being registered as active student in the Dental Faculty of University of Sumatera Utara; (2) no orthodontics treatment or occlusal adjustment history; (3) no facial traumatic injury or drug addiction history. These volunteers were from 18 years to 28 years (mean: 21.61 years ± 2.08 years) old.

**Figure 1:**
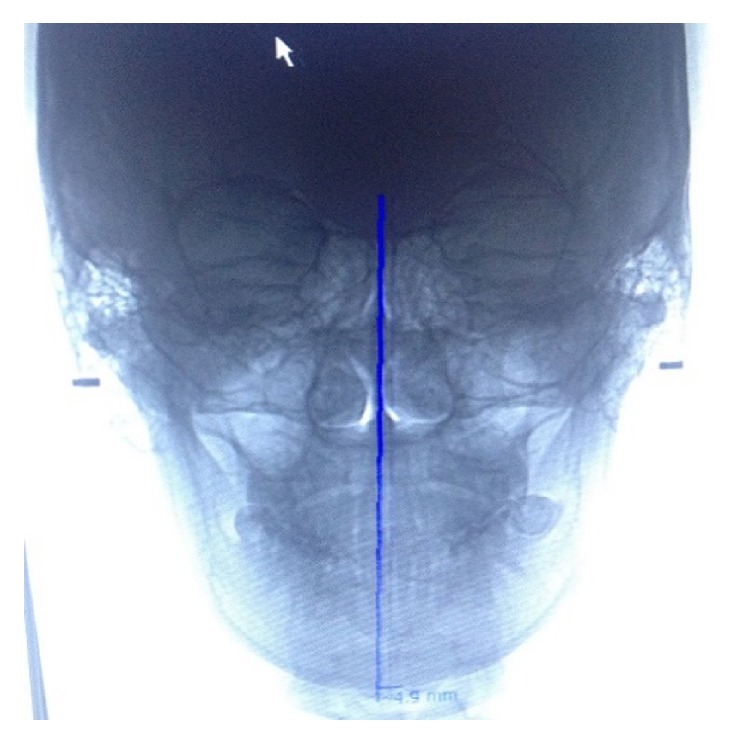
Menton deviation to MSR in PA cephalogram digital radiograph.

The PA digital cephalogram of all the volunteers was taken under standard conditions and processed in the same X-ray machine OC 200 D 1-4-1 with digital sensor in Teaching Hospital Dental Faculty, University of Sumatera Utara, and measured digitally used Cliniview software version 10.1.2. Crista galli (Cg) is establishing in the midline of the skull and located on the midpart of the ethmoid bone which is common to be identified in the PA cephalogram. Menton as the lowest point on the symphyseal shadow of the mandible was reported as one of landmarks that is common in frontal radiographs, such as panoramic and PA cephalogram in mandibular asymmetry [14]. The midsagittal reference (MSR) is constructed from crista galli (Cg) through the Anterior Nasal Spine (ANS) to the chin area. Then the menton deviation was done by measuring the distance of MSR to menton point [4, 15]. If the menton deviation is less than 3 mm, it was categorized as symmetrical group and vice versa if it is more than 3 mm, it was categorized as asymmetrical group (Figure 1()).

Since both TMDs and symmetrical reference used categorize data, there was no normality distribution. The validity and reliability of intrarater digitized cephalometry measurements were obtained by measuring the mean of initial and second measurement and then calculated by using Bland-Altman analysis. The prevalence and amount of menton deviation in PA cephalogram in both groups were evaluated and compared by unpaired *t*-test (SPSS software, version 18.0 for Windows; SPSS, Chicago IL).

## 3. Results

**Figure 2:**
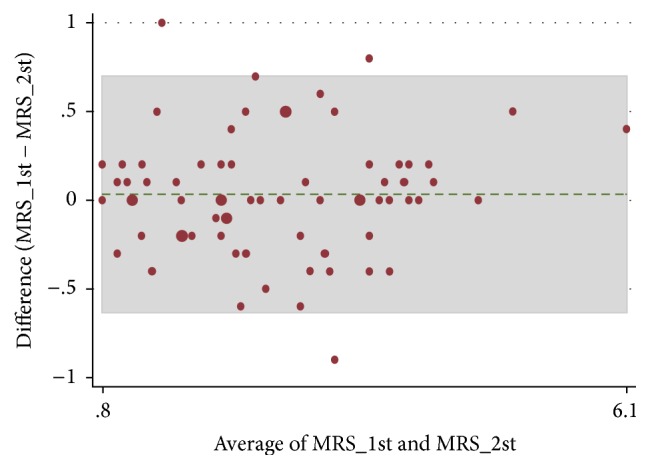
Bland-Altman analysis of combined group.

This study is based on the tracings with intraexaminer reliability of the 70 PA cephalogram of volunteer subjects (mean: 21.61 years ± 2.08 years old) and performed by the same previously trained examiner (40 hours of training). In intraexaminer analysis, the validity and reliability of measurement by quantification of the agreement between two quantitative measurements had constructing limits of agreement of 95% as mean differences of first and second measurement showed no significant difference (*p* = 0.057). There were four measurement samples that showed out of 95% limits of agreement and were eliminated (Figure 2()).

Analysis of unpaired *t*-test (sig. 2 tailed; *p* < 0.05) was used to compare the symmetrical mandible based on menton deviation to TMDs based on TMD-DI questionnaire.

The prevalence of symmetry group showed that 34.1% had TMDs; in contrast, the prevalence of asymmetry group showed that 95.5% reported TMDs in this group. There was a significant difference of menton deviation in both groups. The mean of non-TMDs showed menton deviation 1,815 ± 0,71 mm in non-TMDs and 3,159 ± 1,053 mm in TMDs group (Table 2).

## 4. Discussion

PA cephalogram, which has been reported to be widely used in orthodontics since 1990s, is an important radiodiagnostic in evaluating transverse skeletal and dentoalveolar asymmetry. Vertical and transverse measurement of skeletal and dentofacial structures were obtained relative to the reference lines by comparing the measurements of corresponding structures from the right and left sides. However, there were some limitations in difficulty when reproducing head posture and errors in identifying landmarks [16, 17]. Difficulty in reproducing good head posture might be related to confirming the postural changes of the head and body when taking the PA cephalogram. Adequate head position was required in taking PA cephalogram to avoid bias in measurement. Even though the PA cephalogram procedure was taken, tiny rotation head could affect the MSR analysis [18]. Nowadays, errors in identifying landmarks could be limited by computer-aided cephalometric analysis with digital radiography. Precise written definitions describing the landmarks and clinicians' training before intraexaminer measurement when digitizing landmarks are supposed to reduce the chance of interpretation error [16, 17].

There were several methods for constructing the vertical references lines using anatomic point at crista galli (Cg) to Nasion (Na), Anterior Nasal Spine (ANS), and menton [16]. Even though it was reported that Cg-ANS and Na-ANS had the lowest validity and should not be used in asymmetry cephalometric analysis, our study used an alternative way of constructing the MSR line, which is a line perpendicular to the line connecting the left and right intersection of the zygomaticofrontal suture and lateral orbital margin (ZF-ZF) through the Cg if anatomical variations in the upper and middle facial regions exist [15]. According to Broadbent, menton point is the most inferior point on the symphysis of the mandible in the median plane. There are several types of menton, such as concave type (the highest point between two mental protuberances); convex (the tip of the mandible in the prominent mandible); and flat type (the midpoint of the plat area).

The mandibular deviation resulting in chin deviation towards contralateral side should be considered in orthodontic treatment planning and evaluation of facial asymmetry patients. It means that the menton deviation, maxillomandibular midline angle, and the distance of lower incisor to the midsagittal reference might be compensated by leaning towards the deviated side of the menton during an orthodontic treatment, for example, using elastics [3]. This study used menton point because the chin deviation was easily identified in facial asymmetry patients and reported around 4% in mandibular asymmetry that required orthodontic treatment [19].

Some studies have shown that a small amount of asymmetry in the maxillofacial region is common in general populations and focused on deviation of menton, chin, or gonial angle. The facial asymmetry can be recognized if the menton is deviated by more than 4 mm [3, 15]. Other studies have reported more than 2 mm difference in these points to be recognized as asymmetry [6]. However, this study used 3 mm as the symmetrical guideline based on threshold of visual perception of facial asymmetry in a facial paralysis model that at least 3 mm of the oral commissure, brow, or both was assessed as facial asymmetry [4]. The other consideration in our study was that the subjects were female dental faculty students whose visual perception is more sensitive than layperson. Anamnestic data gathering was conducted according to the TMD-DI which should be considered different with layperson because the subjects of this study were dental faculty students where a person's appearance and self-esteem concern are probably higher than other subjects.

The impact of TMDs was found in some studies showing variation between activities and individuals. It was reported that 52.8% of 142 dental students at Dental school of Casablanca showed at least one sign of TMDs and 17.5% presented with pain [20]. Pain in TMDs has a significant negative impact on activities of daily living, especially to patients with malocclusion [21]. According to Olsson and Lindquist, orthodontic patients appear to be at greater risk of developing TMDs than individuals who only need minor treatment [21]. The presence of postural changes compared between women (mean age 18–45 years old) with migraine with or without TMDs showed clinically relevant postural changes [2]. In our study, the volunteer subjects were female dental student and early detection with TMD-DI questionnaire was performed as initial screening for TMD symptoms. Since we know that TMDs are multifactorial and have been demonstrated to induce mandibular asymmetry, any displacement of anatomical landmark of the mandible might induce skeletal change in the future. Our study was also similar with Purbiati's in adolescent population that reported TMDs as one of the main risk factors of mandibulofacial asymmetry [10]. Those studies indicated the possibility of early detection of TMDs through the presence of facial asymmetry.

Some studies reported that the prevalence of TMDs with various sign and symptoms was higher in older subjects. The prevalence of TMD increases by the age with a mean age of 32.7 ± 14.5 years, while the later comprised mean age of 54.2 ± 15.1 years. The homogenous subjects in age and sex related to the previous studies that reported at least two distinct age peaks are identifiable within this population of patients seeking for TMD treatment, one at about 30–35 years and the other one at about 50–55 years. The ratio of female patients who had sign and symptoms of TMDs was also reported to be higher than male patients. Even though sign and symptom of TMD showed no significant differences in age, sex, and race/ethnicity, the prevalence of female is higher than the male based on age distribution of group diagnoses [1]. There was a deviation of menton from the vertical plane in subjects with TMDs, highlighted by the significant differences of the angle from ANS-Me to the vertical plane among unilateral TMDs, bilateral TMDs, and no TMDs. The asymmetric index of the distances from the vertical plane to the chin or menton point (*p* = 0.02) was higher in subjects with unilateral TMDs [5]. In our study, the prevalence of symmetry group showed that 65.9% had no TMDs; in contrast, the prevalence of asymmetry group showed that 95.5% reported TMDs. There was a significant difference of menton deviation to TMDs (*p* = 0.000) in subjects with and without TMDs in this study. However, our hypothesis that the menton deviation might be useful as symmetrical guideline in early detection of TMDs required larger population sample and approved the clinical examination. The variation of morphological landmarks in PA cephalograms together with functional analysis might be considered as the sign of TMDs.

## 5. Conclusions

Within the limits of the current study, it can be concluded that there was a significant relationship of menton deviation in PA cephalogram with TMD diagnosed by TMD-DI index. Since PA cephalogram analyzes asymmetry cases in skeletal aspect, whereas TMD problem in mandibular asymmetry cases related to the difference of the skeletal measurement and shape of the condyle area, there is a close relationship between mandibular deviation and TMD. A further diagnostic study is needed to confirm PA analysis as an alternative tool for TMD diagnoses.

## Figures and Tables

**Table 1:** Temporomandibular disorder diagnostic index (TMD-DI).

Number	Question lists	Code	Filling instructions
1	Do you have symptom such as headache?		Fill in code with 0 = never 1 = sometimes 2 = often 3 = always
2	Do you have symptom such as pain during closing and opening mouth?		
3	Do you have symptom of joint trismus when getting up in the morning?		
4	Do you have symptom of pain around neck?		
5	Do you have symptom of tinnitus?		
6	Do you clench your teeth in worries?		
7	Do you clench your teeth when in anger?		
8	Do you clench your teeth when concentrating?		

*Total score*			
Total score: 0–24 Total score ≤ 3: TMD symptom code = 0 Total score > 3: TMD code = 1			

**Table 2:** Correlation of menton deviation horizontally to TMDs group (unpaired *t*-test).

Menton deviation	TMD-DI				Mean ± SD	*p* value
	(−)		(+)			
Symmetrical	29	65.9%	15	34.1%	1,815 ± 0,71 mm	0.000^*∗*^
Asymmetrical	1	4.5%	21	95.5%	3,159 ± 1,053 mm	

^*∗*^ *p* < 0.05: significant correlation.
